# Nano/Mesoporous Carbon from Rice Starch for Voltammetric Detection of Ascorbic Acid

**DOI:** 10.3390/molecules23020234

**Published:** 2018-01-25

**Authors:** Mohammad A. Wahab, Farzana Darain, Nazrul Islam, David James Young

**Affiliations:** 1 Faculty of Science, Health, Education and Engineering, University of the Sunshine Coast, Maroochydore DC, QLD 4558, Australia; 2 Australian Institute for Bioengineering and Nanotechnology (AIBN), University of Queensland, St Lucia, QLD 4072, Australia; fdarain@yahoo.com; 3 Sugar Research Australia Ltd., Indooroopilly, QLD 4068, Australia; 4 Pharmacy Discipline, Faculty of Health, School of Clinical Sciences, QUT, 2 George Street, Brisbane, QLD 4000, Australia; nazrul.islam@qut.edu.au

**Keywords:** mesoporous carbon, carbon, rice starch, biomass, electroanalysis, ascorbic acid, electrode, glass carbon electrode, nanomaterials

## Abstract

Rice starch (RS-)based nano/mesoporous carbon (RSNMC) was prepared via a hard-templating route using cheap rice starch as a carbon source. XRD and TEM characterization indicated the formation of organized nanoporous RSNMC. Nitrogen absorption–desorption studies revealed a high surface area of up to 488 m^2^∙g^−1^, uniform pore size of 3.92 nm, and pore volume of 1.14 cm^3^∙g^−1^. A RSNMC-modified glassy carbon (GC) electrode was employed for the determination of ascorbic acid (AA) and exhibited a linear response in the concentration range of 0.005–6.0 mM with a detection limit of 0.003 mM. These results demonstrate that RSNMC has potential as an advanced and cheap electrode material for electrochemical sensing and other electrocatalytic applications.

## 1. Introduction

Nano/mesoporous carbon materials (NMC) are of interest because of their high surface area, uniform pore structure, mechanical strength, chemical stability, and relative lack of reactivity [1,2,3,4,5,6,7]. These characteristics are suited to various applications including as electrodes and support materials, and for catalysis, adsorption, immobilization, energy storage, drug delivery, and electrochemistry [1,2,3,4,5,6,7]. The synthesis of NMC usually involves impregnation of a carbon precursor, commonly sucrose, polypyrole, or furfuryl alcohol, into the pores of mesoporous SBA15 silica in the presence of a catalyst, followed by carbonization at high temperature [1,2,3,4,5,6,7]. A catalytic chemical vapor deposition (CVD) method has also been reported for mesoporous carbon but requires specialized apparatus and harsh conditions [4].

In this context, sustainable nanoporous carbon materials from low-cost biomass have become an important area of investigation [5,6]. Recently, hollow carbon spheres for use in Li–S batteries have been produced using a sustainable approach involving the hydrothermal carbonization of monosaccharides as the carbon precursors and silica nanoparticles as the hard-templates [7]. Similarly, carbon materials with lower porosity have been prepared for the same application by the carbonization of mixed colloidal silica and starch [8]. Liu et al. have reported the generation of micro/mesoporous carbon from chemically modified banana peel for size-selective separation of proteins [9]. It should be noted that carbon material from such biomass requires further chemical activation to create pores, which undoubtedly complicates the process.

In this study, we describe a sustainable and cost-effective one-pot synthesis of rice starch-derived nano/mesoporous carbon material (RSNMC) and its application as an electrode material for the detection of ascorbic acid (AA). Ascorbic acid (vitamin C) is an essential nutrient for living cells and plays an important role in metabolic and cell development processes [10,11,12,13]. It helps develop cells and heal injuries, ulcers, and burns, and is involved in the synthesis of collagen, blood vessels, cartilage, bones, and tendons [10,11,12,13]. It is also an active component in a variety of pharmaceutical dosage forms such as high potency multivitamin supplements for the prevention and treatment of vitamin C deficiency, common colds, mental illnesses, infertility, cancer and AIDS [10,11,12,13]. It is consumed as an antioxidant in a variety of pharmaceutical products, foods, fruits, vegetables, and soft drinks. Different methods have been developed for determination of AA in different matrices, including by titration, UV–vis spectrophotometry, capillary electrophoresis, fiber-optic reflectance spectroscopy, HPLC, thermogravimetry, fluorometry, etc. [13,14,15,16,17,18,19,20,21,22,23,24,25,26,27,28,29]. Electrochemical techniques offer advantages of simplicity, selectivity, stability, and applicability in different matrices. AA is electrooxidized on conventional electrode surfaces; however, this process occurs with high overpotential, which can cause fouling of the electrode surface by its oxidation intermediates or products [22,23]. Thus, these electrodes will have poor reproducibility and reduced selectivity and sensitivity. To overcome these problems, various materials have been immobilized on conventional electrode surfaces, including conducting polymers, ionic liquids, metal nanoparticles, carbon nanotubes, metal complexes, and polymeric films [24,25]. This study reports the use of cheap, nontoxic rice starch-derived nano/mesoporous carbon (RSNMC) as an electrode coating material for the detection of AA using cyclic voltammetry with a RSNMC-modified glassy carbon electrode.

## 2. Experimental

### 2.1. Synthesis of the Hard Mesoporous SBA15 Silica Nano-Template

Surfactant Pluronic P123 (2.0 g) (Sigma-Aldrich, St. Louis, MO, USA) was added to 60 mL of 2 M HCl at 38 °C with vigorous stirring for 2 h. Tetraethylorthosilicate (TEOS) (4.2 g) (Sigma-Aldrich) was added dropwise to the surfactant-containing solution with stirring for another 8 min and left to stand for 24 h at 38 °C. This solution was then autoclaved at 100 °C for 24 h, filtered, and dried at room temperature. Finally, calcination was performed at 550 °C for 6 h in air [30,31,32,33,34,35].

#### 2.1.1. Synthesis of Nano/Mesoporous Carbon (RSNMC) from Rice Starch

**Figure 1:**
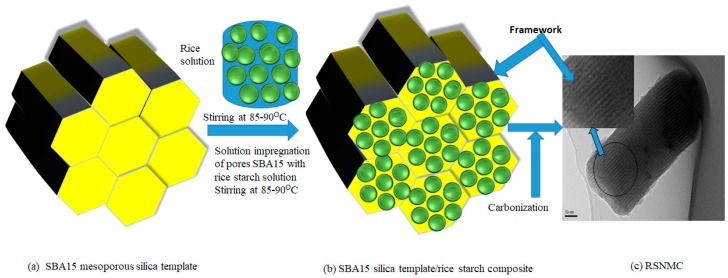
Preparation of nano/mesoporous carbon nanomaterials from rice starch.

Rice starch (2.0 g) (Sigma-Aldrich) in 20 mL of deionized water was stirred in a beaker at 85–90 °C. SBA15 mesoporous silica template (2.3 g) (Figure 1a) was added slowly to the starch solution with vigorous stirring to ensure a homogenous solution of template and rice starch (Figure 1b). Stirring overnight yielded a viscous gel that was collected in a petri dish and dried in an oven at 60 °C for 12 h to remove the solvent, then at 100 °C for 6 h, followed by 160 °C for 6 h. Carbonization was carried out under an argon flow at 900 °C for 1 h The silica template was removed by washing with 5 wt % HF solution at room temperature for 6 h [30,31,32,33,34,35]. The resulting nano/mesoporous carbon (Figure 1c) was collected by filtration, washed with ethanol, and dried in an oven at 100 °C.

#### 2.1.2. Electrode Preparation and Electrochemical Measurements

The RSNMC was predried at 60 °C for 1 h in a conventional oven. RSNMC (5 mg) was added to 20 mL of ethanol and sonicated for 30 min to obtain a well-dispersed, homogeneous suspension. The bare glassy carbon (GC) electrode was polished with 0.50 and 0.05 μm alumina slurry and rinsed with water. It was then sonicated in deionized water for 5 min and dried under a high-purity nitrogen stream. The RSNMC ethanol suspension (4 µL) was drop-cast onto the cleaned GC electrode and dried under a nitrogen atmosphere at room temperature to obtain the RSNMC/GC electrode.

Electrochemical experiments were performed on a Bioanalytical Systems BAS 100B/W electrochemical workstation with three electrodes including the RSNMC-modified GC working electrode, an Ag/AgCl reference electrode, and a platinum wire counter electrode. A 0.1 M PBS, pH 7.0 solution was used as the supporting electrolyte for the potential cycling of potassium ferricyanide, K_3_Fe(CN)_6_, and AA. Electrochemical measurements were performed under a Nitrogen atmosphere using solutions deoxygenated by purging with N_2_ for about 15 min. All measurements were carried out at room temperature (25 °C).

#### 2.1.3. Characterization

X-ray diffraction (XRD) patterns were recorded using a Bruker radiation D8 Advanced Diffractometer with Cu Kα radiation for angles 1 to 10 2θ (degrees). A Rigaku Miniflex diffractometer (Japan) with Cu Kα radiation was used for the 2θ range from 10 to 80° under identical conditions at a scanning rate of 2 degrees. An automated adsorption analyzer (Quadrasorb SI, Quantachrome, Boynton Beach, FL, USA) was used to study the Brunauer–Emmett–Teller (BET) surface areas, pore sizes, and pore volumes. All samples were outgassed at 180 °C for 12 h under vacuum prior to analysis using the degas port of the adsorption analyzer. Pore size distribution (PSD) was obtained using the Barrett–Joyner–Halenda (BJH) model from the desorption branch of the isotherm [36,37]. The pore morphologies were observed by ultrasonically dispersing the sample in ethanol for 10 to 15 min and then casting onto carbon-coated Cu grids using a dropper. TEM (TEM, FEI Tecnai 20, 200 kV) images were obtained on an F20 microscope with an accelerating voltage of 200 kV. Solid-state NMR spectra were acquired on an Avance III spectrometer (Bruker), operating at 59.627 MHz for ^29^Si and 75.468 MHz for ^13^C. Powdered material was placed in the 4 mm zirconium rotor and rotated at the magic angle at 7 kHz. Spectra were acquired using a 42 ms acquisition time, sweep width of 50 kHz, 2 K data points, and high-power ^1^H TPPM decoupling. Recycle times ranged from 30 s to 100 s and were verified to be sufficient for relaxation without signal saturation. ^29^Si NMR spectra were recorded after a single pulse to allow for quantification of the Si species. The 90° pulse was followed by high-power ^1^H TPPM decoupling at 87 kHz. X-ray photoelectron spectroscopy (XPS) was performed using a Kratos Axis ULTRA X-ray photoelectron spectrometer equipped with a 165 mm hemispherical electron energy analyzer using a monochromatic Al Kα (1486.6 eV) X-ray source at 150 W (15 kV, 10 mA). A survey wide scan was collected at an analyzer pass energy of 160 eV and multiplex (narrow) high-resolution scans of 20 eV. The base chamber pressure was 1.0 × 10^–9^ Torr, increasing to 1.0 × 10^–8^ Torr during sample analysis. The binding energies were referenced to the C 1 s peak of adventitious carbon at 284.6 eV to account for charging effects.

## 3. Results and Discussion

**Figure 2:**
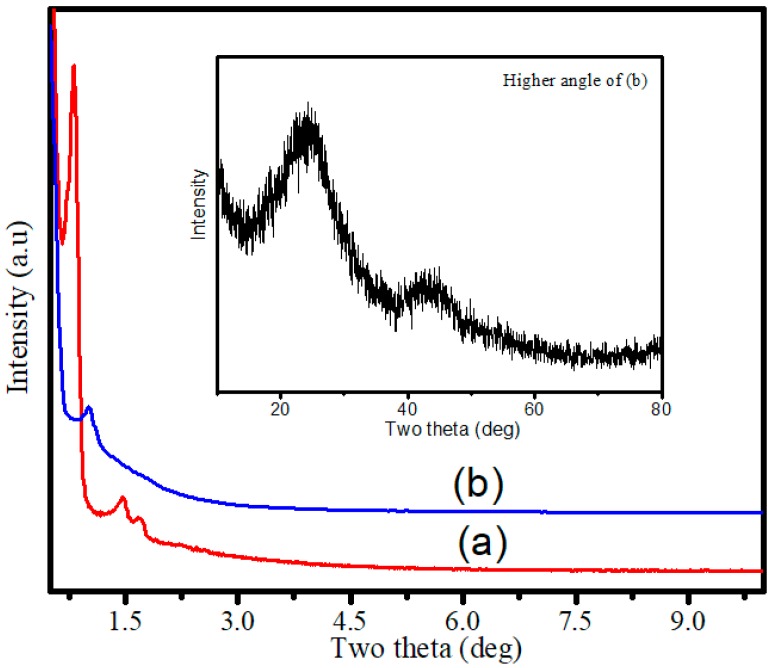
(**a**) XRD patterns of calcined SBA-15 silica template and (**b**) the rice starch-based nano/mesoporous carbon (RSNMC). The inset shows the higher angle XRD pattern of the RSNMC.

**Figure 3:**
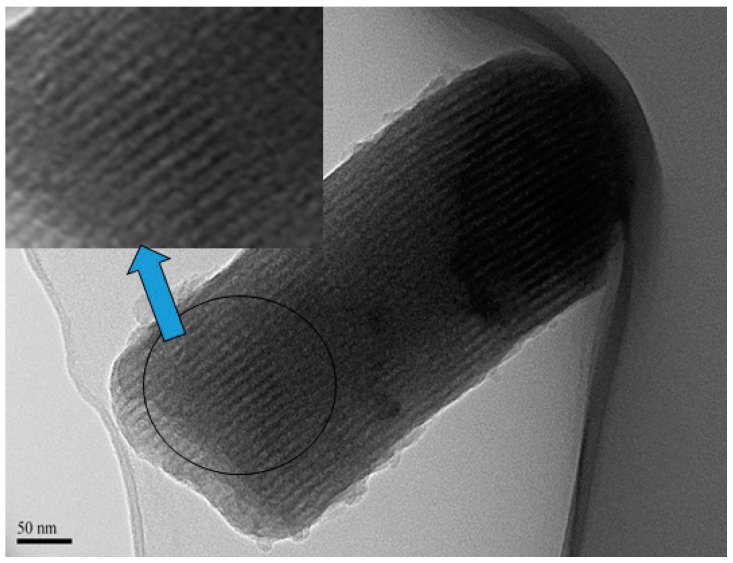
Transmission electron microscope (TEM) image of RSNMC.

RSNMC was prepared using a nanoporous SBA silica template (Figure 1). The high surface area, large pore size, and pore volume of the hard template were impregnated with the rice starch precursor solution at 85–90 °C. The rice starch precursor was carbonized at a high temperature and the template was removed by washing with aqueous HF solution. The XRD pattern of the SBA15 mesoporous silica template (Figure 2a) showed three well-resolved XRD peaks at low angles that could be indexed as (100), (110), and (200) reflections, indicating the formation of well-ordered hexagonal materials (p*6*mm) [3,30,31,32,33,34,35]. The XRD pattern of the corresponding RSNMC material (Figure 2b) exhibited a sharp peak at 2*Θ* = 1.035 with a secondary hump-like reflection consistent with the formation of mesoporous carbon [3,30,31,32,33,34,35,36,37,38,39,40,41,42]. The (100) peak at 2*Θ* = 1.035 for the RSNMC exhibited a d-spacing of 8.53 nm, which corresponds to a unit cell parameter of 9.85 nm, whereas the two broad peaks at wide-angle XRD patterns (Figure 2 inset) suggest the formation of an amorphous, graphitic carbon framework. The unit cell parameter of the RSNMC replica is smaller than that of the SBA-15 template, presumably due to shrinkage during carbonization and dissolution of the host silica framework. Pore formation in the RSNMC was investigated by TEM (Figure 3) and indicated organized nanopore channels [3,30,31,32,33,34,35,36,37,38,39,40,41,42].

**Figure 4:**
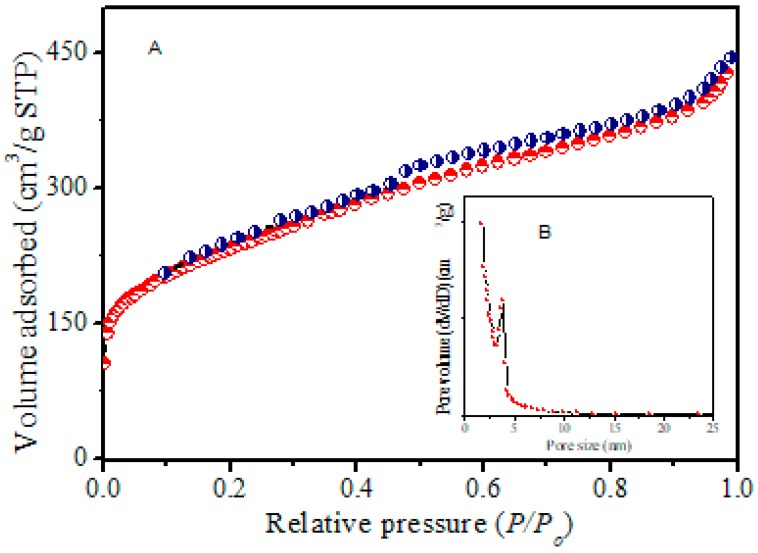
(**A**) N_2_ isotherms of rice starch-based mesoporous carbon RSNMC; (**B**) The inset shows the pore size distribution of RSNMC.

BET isotherms (Figure 4) from N_2_ adsorption–desorption measurements exhibited well-developed type IV hysteresis curves in the range of 0.4 to 0.75, indicating the formation of mesoporous carbon nanostructures [3,30,31,32,33,34,35,36,37,38,39,40,41,42]. The corresponding BJH pore size distribution (Figure 4 inset) displayed a pore size centered at about 3.92 nm in size. A surface area of 488 m^2^∙g^−1^, total pore volume of 1.14 cm^3^∙g^−1^, and pore size of 3.92 nm were determined as shown in Table 1. Overall, results are consistent with previously reported mesoporous structured materials [3,30,31,32,33,34,35,38,39,40,41,42,43].

XPS was used to obtain further information about the elemental composition of the RSNMC. An XPS survey spectrum (Figure SI 1) clearly shows two peaks at ~284.08 and ~530.3 eV, which are responsible for carbon and oxygen, respectively [3,39,40,41,42]. The C1s peak is much stronger than the O1s peak, reflecting their relative abundance. The single intense C peak at 284.08 eV could be assigned to a pure carbon environment whereas the little broad peaks at about 286.2 eV, and 290.2 eV, correspond to C–O and C=O moieties, respectively [3,38,39,40]. Another very minor peak at 530.3 eV is due to the oxygen content that remained in the mesoporous carbon framework [3,39,40,41,42]. Solid-state ^29^Si NMR spectra (Figure SI 2) confirmed the efficient removal of the silica template by HF treatment [44]. The ^13^C CP-MAS NMR spectrum of the resulting RSNMC sample indicates (Figure SI 3) a predominance of sp^2^ rather than sp^3^ carbons, consistent with a graphitic carbon framework [42,43,44].

## 4. Electrochemical Behavior of RSNMC

**Figure 5:**
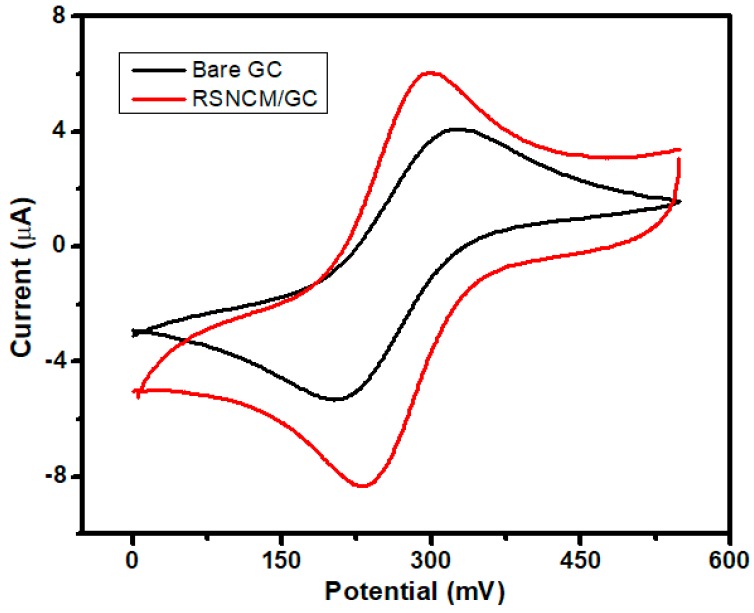
CVs obtained for a RSNMC/ glassy carbon (GC) and bare GC electrode in 1.0 mM K_3_Fe(CN)_6_ prepared in PBS, pH 7.0 at scan rate of 20 mV/s.

The electrochemical performance of RSNMC as a novel electrode material was evaluated using potassium ferricyanide (1 mM K_3_[Fe(CN)_6_] prepared in PBS, pH 7.0). The cyclic voltammogram (CV) curves obtained by using a RSNMC-modified GC electrode and a bare GC electrode at a potential sweep rate of 20 mV/s are shown in Figure 5. The two CVs show similar redox peak behavior. Indeed, the redox peak of potassium ferricyanide at the RSNMC/GC electrode is much higher than that obtained at the bare GC electrode, indicating that the former possesses a larger electrode area relative to the bare GC electrode. The electroactive surface area of both electrodes was obtained from the Randles–Sevcik equation [45]:

iP = (2.69 × 10^5^) AD^1/2^n^3/2^v^1/2^c* (1)

where iP is the peak current (A), n is the number of electrons participating in the redox reaction, A is the electroactive surface area (cm^2^) of the electrode, D is the diffusion coefficient of [Fe(CN)_6_]^3−^ (taken to be 7.60 × 10^−6^ cm^2^∙s^−1^ in aqueous medium), c* corresponds to the bulk concentration of the redox probe (mol∙cm^−3^), and v is the scan rate (V/s). The calculation indicates an electroactive surface area for RSNMC/GC electrode of 0.052 cm^2^, which is 1.7 times higher than that of a GC electrode (0.03 cm^2^). In addition, the peak potential separation (Δ*Ep*) between the anodic and cathodic peaks is 57 mV for the RSNMC/GC electrode and 103 mV for the GC electrode. The decrease in Δ*Ep* indicates that the RSNMC/GC electrode not only possesses a high surface area, but also accelerates the electron transfer rate of ferricyanide [13,28]. This experiment suggests that the presence of RSNMC could increase the relative electron transfer of the GC electrode [13,28,29].

**Figure 6:**
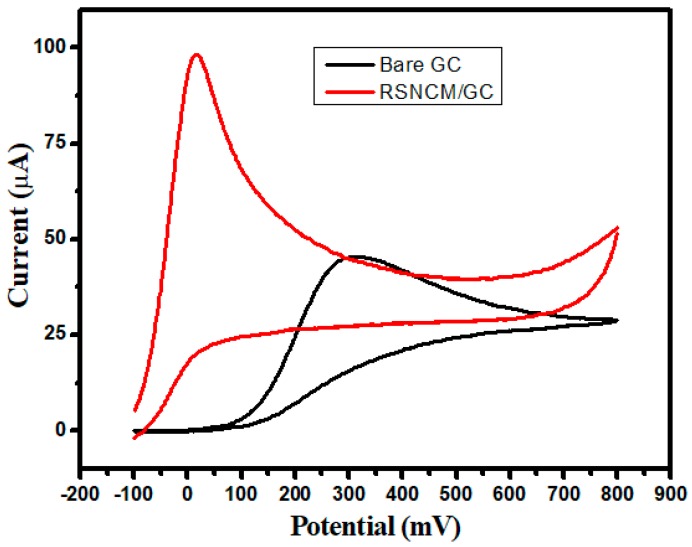
CVs obtained for RSNMC/GC and bare GC in 3 mM ascorbic acid prepared in PBS, pH 7.0 at scan rate of 20 mV/s.

The CV response of an RSNMC-modified GC electrode towards the oxidation of ascorbic acid was investigated and the result was compared to that of an unmodified GC electrode. This oxidation process was irreversible as shown in Figure 6. A well-defined oxidation peak was observed with significant current enhancement and peak potential shift of ~300 mV in the negative direction relative to an unmodified GC electrode. The electron transfer ability of ascorbic acid was increased for the RSNMC-modified GC electrode. It is clear for this experiment that the oxidation of ascorbic acid was greatly enhanced by the use of RSNMC as the electrode material. This superiority in analytical performance can be ascribed to the high surface area, uniform and larger pore volume, and surface roughness of the interface [13,28,29].

**Figure 7:**
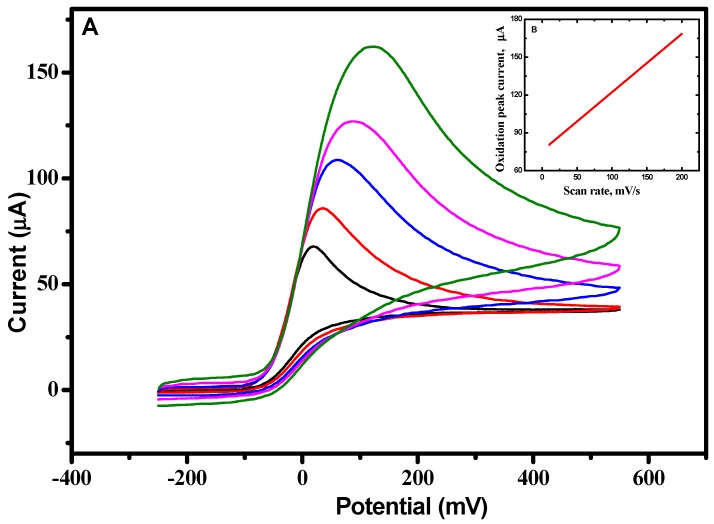
(**A**) CVs were obtained at a RSNMC/GC electrode in 3 mM Ascorbic acid prepared in PBS, pH 7.0 at 10, 20, 50, 100, and 200 mV/s scan rates; and (**B**) relationship between peak current and scan rate.

**Figure 8:**
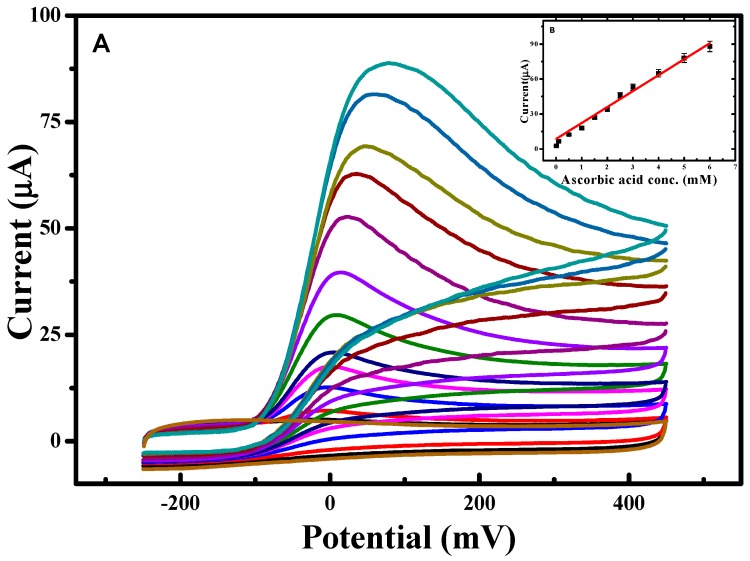
(**A**) CVs obtained at various concentrations of ascorbic acid prepared in PBS, pH 7.0 at scan rate of 10 mV/s; and (**B**) calibration plot showing concentration of ascorbic acid prepared in PBS, pH 7.0 at scan rate of 10 mV/s.

Systematically changing the scan rate (Figure 7) to investigate the diffusion behavior revealed that the anodic peak potential was shifted in the positive direction with a concomitant, linear increase in the oxidative peak current (Figure 7B). The slope of this plot was ~0.5, indicating that the current is primarily diffusion controlled [14]. Figure 8 illustrates the dependence of the CV response using the RSNMC/GC electrode to the addition of different concentrations of ascorbic acid ranging from 0.005 to 6.0 mM in PBS, pH 7.0. This plot exhibited good linearity described by the equation *y* = 1.679*x* + 8.594 with an *R^2^* value of 0.997, and allowed calculation of a detection limit of 0.003 mM, which is lower than those of a multi-walled titanium oxide composite-modified GC electrode and a single-walled carbon nanotube zinc oxide-modified GC electrode, and similar to that of a reduced graphene oxide–cobalt hexacyanoferrate nanocomposite electrode [13,28,29].

The reproducibility of our RSNMC/GC electrode was evaluated by repetitive electrochemical measurements in a solution containing 3 mM ascorbic acid. The modified electrode possessed a relative standard deviation of around 3.0% after 10 consecutive measurements. In addition, five freshly prepared RSNMC-coated GC electrodes were used to detect ascorbic acid in PBS. All electrodes exhibited similar current responses with a relative standard deviation of 3.0%. Finally, an electrode was stored in a desiccator for nearly four weeks after use and retained its electrochemical reactivity.

## 5. Conclusions

In summary, we have successfully demonstrated that rice starch can be used as an inexpensive biomass precursor source for nano/mesoporous carbon (RSNMC). Carbonaceous nanostructures were prepared using a hard, mesoporous silica template and exhibited a high surface area (488 m^2^∙g^−1^), large pore volume (1.14 cm^3^∙g^−1^), and narrow pore size distribution (3.92 nm) without requiring chemical activation. A RSNMC-modified GC electrode displayed enhanced electrocatalytic activity towards the electrochemical oxidation of ascorbic acid as indicated by current enhancement and an associated peak shift compared with an unmodified GC electrode. The constructed porous carbon interface displayed strong signal amplification. The RSNMC-modified GC electrode demonstrated a reproducible, linear relationship for ascorbic acid detection over the range 0.005–6.0 mM and a detection limit of 0.003 mM. The preparation of RSNMC is very straightforward and our results indicate that it has superior performance as an electrode material.

## Figures and Tables

**Table 1:** Surface structural properties of pure SBA15 silica template and RSNMC.

Sample	BET Surface (m^2^/g)	Pore Size (nm)	Pore Volume (cm^3^/g)
SBA15 silica template	915	9.15	1.12
RSNMC	488	3.92	1.14

## Supplementary Materials

Supplementary materials are available online.
