# Supplementary material for: Nano/Mesoporous Carbon from Rice Starch for Voltammetric Detection of Ascorbic Acid
Source: Molecules. 2018 Jan 25;23(2):234. doi: 10.3390/molecules23020234 (PMC6017438; doi:10.3390/molecules23020234)
Supplement: Supplementary file 1 [file molecules-23-00234-s001.pdf]

Supporting information

Nano/mesoporous Carbon from Rice Starch for Voltammetric Detection of Ascorbic Acid

Md A Wahab,[ab*Π] Farzana Darain[bdΠ], Nazrul Islam,[c] and David James Young[a*]

[abΠ] Faculty of Science, Health, Education and Engineering, University of the Sunshine Coast, Maroochydore DC, Queensland 4558, Australia; [c]Pharmacy Discipline, Faculty of Health, School of Clinical Sciences, QUT, 2 George Street, Queensland, Australia

[b]AIBN, University of Queensland, Australia

[dΠ]Sugar Research Australia Ltd., Indooroopilly, Brisbane, Australia.

[Π] = Current affiliation and equal contributor

*Corresponding E-mails: mawahab@gmail.com; mwahab@usc.edu.au; dyoung1@usc.edu.au

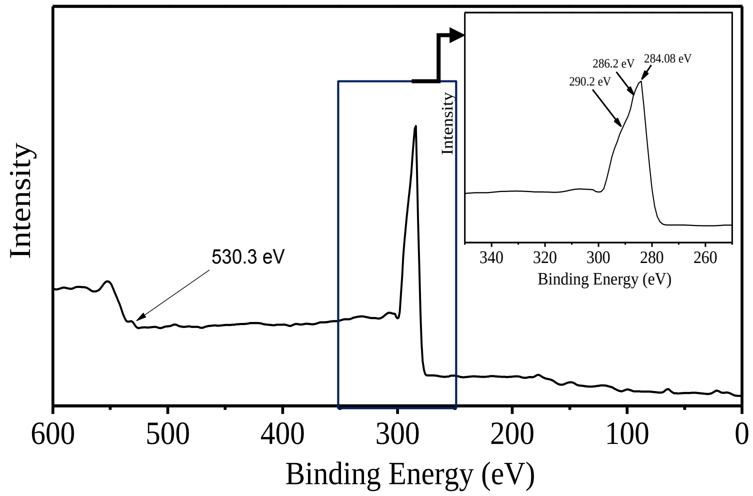

Figure SI 1. XPS data of RSNMC material.

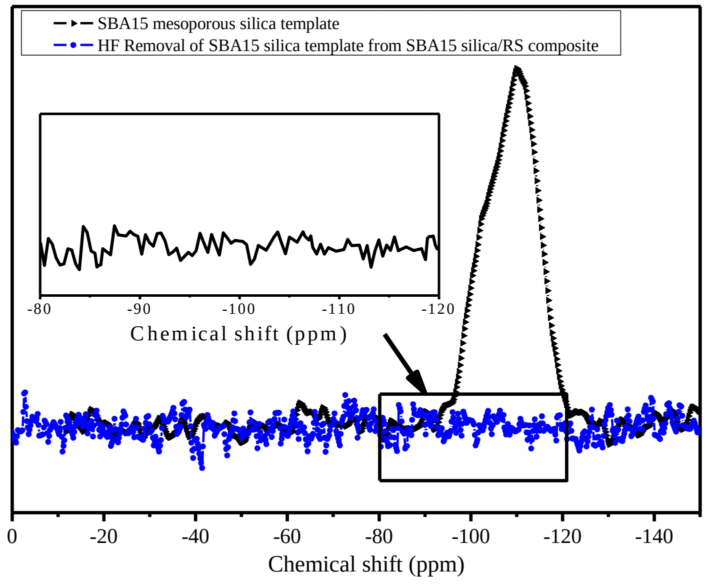

Figure SI 2. SBA15 silica removal by HF washing.

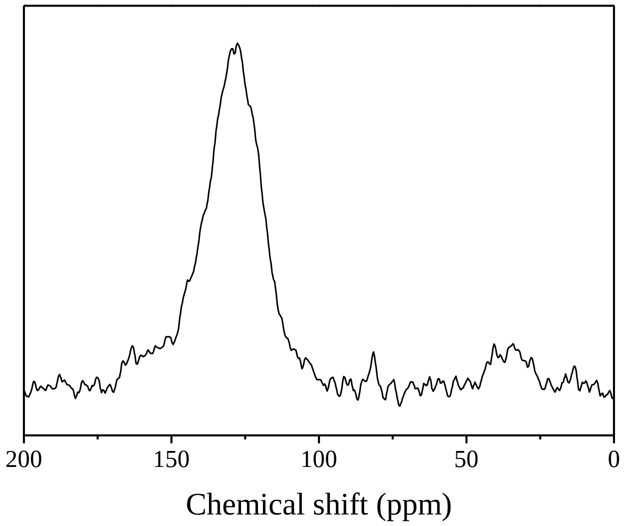

Figure SI 3. 13C NMR spectrum of RSNMC after removing SBA15 silica template
